# Strain-Dependent Induction of Human Enterocyte Apoptosis by *Blastocystis* Disrupts Epithelial Barrier and ZO-1 Organization in a Caspase 3- and 9-Dependent Manner

**DOI:** 10.1155/2014/209163

**Published:** 2014-04-14

**Authors:** Zhaona Wu, Haris Mirza, Joshua D. W. Teo, Kevin S. W. Tan

**Affiliations:** Laboratory of Molecular and Cellular Parasitology, Department of Microbiology, Yong Loo Lin School of Medicine, National University of Singapore, 5 Science, Drive 2, Singapore 117596

## Abstract

*Blastocystis* is an emerging protistan parasite colonizing the human intestine. It is frequently reported to cause general intestinal symptoms of vomiting, diarrhea, and abdominal pain. We recently demonstrated that *Blastocystis* rearranged cytoskeletal proteins and induced intestinal epithelial barrier compromise. The effect of *Blastocystis* on enterocyte apoptosis is unknown, and a possible link between microbially induced enterocyte apoptosis and increased epithelial permeability has yet to be determined. The aim of this study is to assess if *Blastocystis* induces human enterocyte apoptosis and whether this effect influences human intestinal epithelial barrier function. Monolayers of polarized human colonic epithelial cell-line Caco-2 were incubated with *Blastocystis* subtype 7 and subtype 4. Assays for both early and late markers of apoptosis, phosphatidylserine externalization, and nuclear fragmentation, respectively, showed that *Blastocystis* ST-7, but not ST-4, significantly increased apoptosis in enterocytes, suggesting that *Blastocystis* exhibits host specificity and strain-to-strain variation in pathogenicity. ST-7 also activated Caco-2 caspases 3 and 9 but not 8. ST-7 induced changes in epithelial resistance, permeability, and tight junction (ZO-1) localization. Pretreatment of Caco-2 monolayers with a pan-caspase inhibitor z-VAD-fmk significantly inhibited these changes. This suggests a role for enterocyte apoptosis in * Blastocystis*-mediated epithelial barrier compromise in the human intestine.

## 1. Introduction

*Blastocystis *is an anaerobic protistan parasite of the gut [1–3]. It is a species complex comprising of 17 subtypes out of which at least 9 are known to infect humans [4]. Other common hosts are rats, pigs, and chickens [1]. It is one of the most common parasites found in humans [3] with prevalence ranging from 10% in developed countries to 50% in developing countries [3]. Infection with the parasite is associated with common intestinal symptoms of mucous and watery diarrhea, bloating, abdominal pain, and vomiting [3]. It has a higher prevalence in impoverished children [5] and patients immunocompromised due to HIV infection [6] or malignancy [7], suggesting opportunistic pathobiology [3]. It is often associated with irritable bowel syndrome [8–11] and urticaria [12, 13]. It is also considered one of the causes of traveler's diarrhea [14]. Chronic and recurrent infections are common despite prolonged antimicrobial treatment [13, 15]. Recent studies suggest that in some cases the parasite is also capable of epithelial invasion [16–19].

Despite being discovered more than 100 years ago [20, 21] as well as the recent advances in our understanding of this organism regarding its potential virulence factors and host responses [15, 22], its pathogenic potential remains controversial [4], owing to frequent reports of asymptomatic carriage, nonresponsiveness to chemotherapy, coinfections with other known pathogens, and high degree of genetic and pathobiological diversity within and across subtypes [23]. Strain-to-strain variation in virulence of the parasite has been reported in clinical and animal infection studies [8, 24, 25]. We recently reported that a subtype-7 (ST-7) isolate of* Blastocystis* recovered from a symptomatic patient exhibits higher cysteine protease and arginase activity (potential parasite virulence factors) compared to ST-4 isolates [15, 26]. ST-7 strain also exhibited higher immune evasion potential in inducing degradation of secretory IgAs and downregulation of epithelial antiparasitic nitric oxide production [15, 27]. However, direct evidence of comparative study in pathogenic effects of different* Blastocystis *strains on the host cells with a well-recognized host-pathogen interaction mode is still limited.

Modulation of intestinal epithelial barrier function is one of the major mechanisms employed by pathogens to induce host pathology [28]. Organisms ranging from viruses [29] to bacteria [30] as well as parasites such as* Giardia *[31] and* Entamoeba *[32] are known to compromise epithelial barrier function. Compromise of epithelium's barrier function exposes subepithelial tissue to toxic luminal contents [33], which could lead to dire consequences for the host. Interestingly, various luminal parasites sabotage this gate function by utilizing the apoptotic machinery of the host cell [34, 35]. Apoptosis or programmed cell death is a mechanism of clearing up unwanted cells by the body while simultaneously limiting overt immune response [36].* Entamoeba *induces enterocyte apoptosis to facilitate the parasite infection of gut [37] while* Giardia* induces epithelial barrier compromise by activating caspase 3-mediated enterocyte programmed cell death [38]. There is ample evidence suggesting that* Blastocystis* causes intestinal epithelial barrier compromise in vitro and in vivo [22, 25, 39]. Rodent* Blastocystis *has been shown to induce caspase-mediated apoptosis, with no effect on the barrier function of rat intestinal epithelium [39]. An association of parasite-induced apoptosis with disruption of epithelial barrier function, as observed in* Giardia *and* Entamoeba *infections, is not known in human intestinal epithelium.

By using a human intestinal Caco-2 cell line that has been well established as an in vitro model system to study* Blastocystis*-host interaction [4, 15, 22], we report for the first time that* Blastocystis* subtype 7 (ST-7; isolate B), recovered from a symptomatic patient, induces breakdown of epithelial barrier function and caspase-3- and 9-mediated apoptosis and rearrangement of the tight junction associated protein ZO-1. In contrast, a subtype recovered from a rat (ST-4; isolate WR-1), previously reported to induce rat epithelial injury [39], did not cause any pathology in human epithelium. In this study, caspase inhibition in human gut epithelium prevented the effect of parasite on barrier dysfunction. This contrasts with the previous study involving rat epithelial cells, in which caspase inhibition did not rescue cells from barrier compromise [39].

## 2. Materials and Methods

### 2.1. Culture of Caco-2 Colonic Epithelial Cell Line

All* Blastocystis*-host interaction experiments were performed using Caco-2 human colonic cell line (ATCC). Caco-2 stock cultures were maintained in T-75 flasks in a humidified incubator with 5% CO_2_ at 37°C. Cell cultures were grown in Dulbecco's modified Eagle's medium (HyClone) supplemented with 10% heat-inactivated fetal bovine serum (HyClone) and 1% each of sodium pyruvate, MEM, and antibiotic “Penstrep” (Gibco). Culture health was evaluated using the Trypan blue assay and only cultures with >95% viability were used for the experiments. Cells were trypsinized with 0.25% trypsin-EDTA. Cell cultures for western blotting experiments were grown on standard cell-culture 6-well plates (Corning). For confocal imaging, cells were cultured on poly-L-lysine treated 12 mm glass coverslips, placed in standard 6-well culture plates (Corning). Cell cultures for annexin-V-FITC apoptosis assay were grown on standard 24-well cell-culture plates (Corning). For transepithelial resistance (TER) and permeability experiments, cells were grown on Millipore transwell filters with PET membranes of 3 *μ*m pore size placed in 24-well tissue culture plates. In order to synchronize cells before experiments, all cultures were serum-starved overnight in antibiotic free and serum free DMEM. For caspase inhibition experiments, cell cultures were pretreated with 40 *μ*M broad-spectrum caspase inhibitor, z-VAD-fmk (Sigma) for 4 h. Cytochalasin D (Sigma) was used as a positive control for epithelial resistance and permeability experiments at a concentration of 1 *μ*g/mL. Staurosporine was used as a positive control for apoptosis and ZO-1 rearrangement experiments at a concentration of 0.5 *μ*M.

### 2.2. Parasite Culture and Lysate

Two axenized* Blastocystis* isolates belonging to different subtypes (ST) were used in this study. Isolate B, belonging to ST-7, was isolated from a symptomatic patient at the Singapore General Hospital [26], while isolate WR-1, belonging to ST-4, was isolated from a Wistar rat during an animal survey [26]. Both ST-7 and ST-4 represent zoonotic subtypes. They are frequently isolated from stool samples. ST-7 is often associated with intestinal symptoms and has been known to induce epithelial barrier disruption [8, 22]. Other than humans, common hosts for ST-7 and ST-4 isolates are birds and rats, respectively [40].

Parasite cultures were maintained in prereduced Iscove's modified Dulbecco's medium (IMDM) supplemented with 10% heat-inactivated horse serum. Cultures were kept under anaerobic conditions in an Anaerojar (Oxoid) with gas pack (Oxoid) at 37°C. Only log-phase cultures were harvested for lysate preparation. The cultures were washed twice in sterile PBS. Parasitic lysates were prepared by three freeze-thaw cycles in liquid nitrogen and 37°C water bath. Unless otherwise indicated, monolayers in all experiments were incubated with 10^8^ parasite/mL lysate.

### 2.3. Epithelial Resistance

Transepithelial resistance (TER) across Caco-2 monolayer was measured using Millipore-ERS-2 Volt-Ohm-Meter. Caco-2 monolayers were grown on Millipore transwell system as described above and TER was measured every alternate day until it peaked (~ day 21; 1000 Ω/cm^2^).* Blastocystis* ST-7 and* Blastocystis* ST-4 were coincubated with epithelium for 3, 6, 12, and 24 h. For dose-dependent experiments, monolayers were coincubated with 0.25 to 2 × 10^8^ parasites/mL for 24 h. After incubation, monolayers were carefully washed twice with Hank's balanced salt solution (HBBS). 200 *μ*L of warm media was added to the apical compartment before taking TER measurements. All measurements were done at 37°C to minimize reading fluctuations.

### 2.4. Epithelial Permeability

Caco-2 monolayers were cultured on a transwell system as described earlier till they reach confluency and tight junction maturation on day 21. After confirmation of maturation by TER measurement, monolayers were coincubated with parasites for 24 h. Following coincubation, epithelial and basolateral compartments were washed twice, followed by addition of 400 *μ*L of warm HBSS at the basolateral compartments and 200 *μ*L of 100 *μ*g/mL FITC-conjugated Dextran 4000 solution in HBSS to apical compartments. After 3 h, Dextran-FITC flux across monolayers was measured by transferring 300 *μ*L of basolateral HBSS to a 96-well plate (corning) and measuring fluorescence using an ELISA reader (Tecan Infinite M200) at excitation and emission wavelengths of 492 and 518, respectively.

### 2.5. Flow Cytometry

Annexin-V binding assay was used to observe early apoptotic changes in Caco-2 cells. Caco-2 monolayers were grown in 24-well culture plates and coincubated with* Blastocystis* ST-7 or ST-4 for 3 h. Monolayers were then washed with PBS twice and then were trypsinized with 0.25% trypsin-EDTA, resuspended, and collected. Annexin-V-FITC apoptosis detection kit (BioVision) was used according to manufacturer's instructions. Propidium iodide (PI) was used to exclude necrotic cells. After cell staining, samples were analyzed using a flow cytometer (DakoCytomation; Cyan LX) at 488 nm excitation wavelength, with a 515 nm band-pass filter for fluorescein detection and a 600 nm filter for PI detection. The lower right quadrant was defined to represent the apoptotic cells showing annexin-V-FITC-positive and PI-negative staining.

### 2.6. Immunohistochemistry and Confocal Microscopy

Monolayers were incubated with* Blastocystis *for 6 h for immunohistochemical detection of ZO-1 rearrangements in Caco-2 cells. After coincubation, monolayers were washed twice and fixed with 2% (w/v) formaldehyde in PBS. Cells were then washed and incubated overnight with 1000 × dilution of primary antibody against ZO-1 tight junction protein (Sigma; 1 : 1000 in PBS) at 4°C. Monolayers were washed twice with PBS and incubated for 1 h with Cy3-tagged secondary antibody, followed by another round of washing. For DNA staining assay, monolayers were treated with parasites for 24 h, washed, and fixed as described above. After fixation, monolayers were washed and incubated with 10 *μ*g/mL of cell-permeable DNA-stain Hoechst (Invitrogen) for 10 min and washed again. All monolayers were mounted on a glass slide using fluorescence mounting media (VECTASHIELD) before being observed under a confocal microscope (Olympus BX60; Olympus, Japan). The ImageJ software was used for image analysis.

### 2.7. Western Blots

For western blot analysis, Caco-2 monolayers were grown on 6-well cell-culture plates (corning) until 100% confluency. Monolayers were then incubated with 1 × 10^8^ parasites/mL for 6 and 12 h, respectively, washed with PBS twice, and then scraped and collected. Monolayers were then incubated with RIPA lysis buffer supplemented with protease and phosphatase inhibitors (Pierce). Lysed samples were centrifuged at 21,000 rpm at 4°C for 30 minutes. Protein concentration of the supernatant was determined with the D_C_ Protein Assay (Bio-Rad Laboratories). SDS-PAGE gels (12% and 15% Tris-HCL Ready-Gels; Bio-Rad Laboratories) were used to separate total proteins. Proteins were then transferred using a polyvinylidene difluoride (PVDF) membrane (Immobilon-P; Millipore). 5% of nonfat dry milk in % TBS-T was then used to block the membranes. After blocking, membranes were incubated with primary antibodies against, caspase 3, caspase 8, caspase 9, or ZO-1 (1 : 1000; Sigma) overnight at 4°C. After incubation with primary antibodies, membranes were washed and incubated with HRP-tagged secondary antibodies. Bands were detected using Amersham ECL Plus western blotting detection system (GE Healthcare). Autoradiographic films (Kodak) were then exposed to the membranes and developed on* X-ray* film processor SRX-101A (Konica Minolta).

### 2.8. Statistical Analysis

The ANOVA test was used to confirm the statistical significance of our results.

## 3. Results

### 3.1. *Blastocystis* ST-7 Decreases Transepithelial Resistance (TER) and Increases Permeability to FITC-Conjugated Dextran in Caco-2 Monolayers

**Figure 1:**
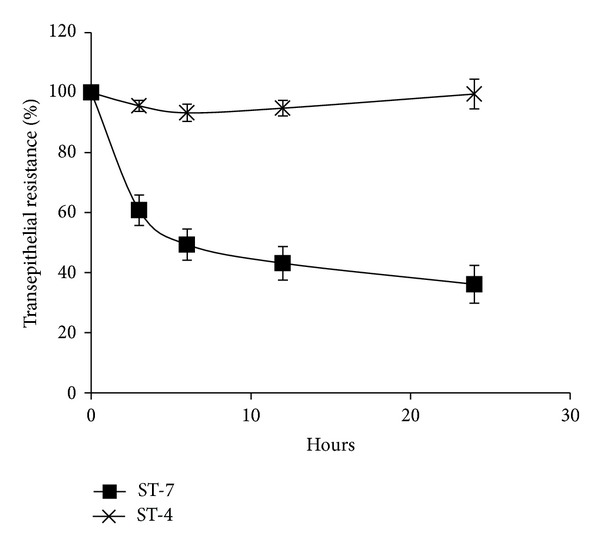
Time-dependent effect of* Blastocystis *ST-7 and ST-4 on the transepithelial resistance (TER) of Caco-2 cell monolayers. Confluent monolayers of Caco-2 cells were coincubated with ST-7 and ST-4 for the indicated times. TER was then measured as described in Section 2. ST-7 treated monolayers, compared to ST-4 and negative control, showed a significant drop in TER after 3, 6, 12, and 24 h (*P* value < 0.01). Values are means ± standard error (error bars) (*n* = 6).

**Figure 2:**
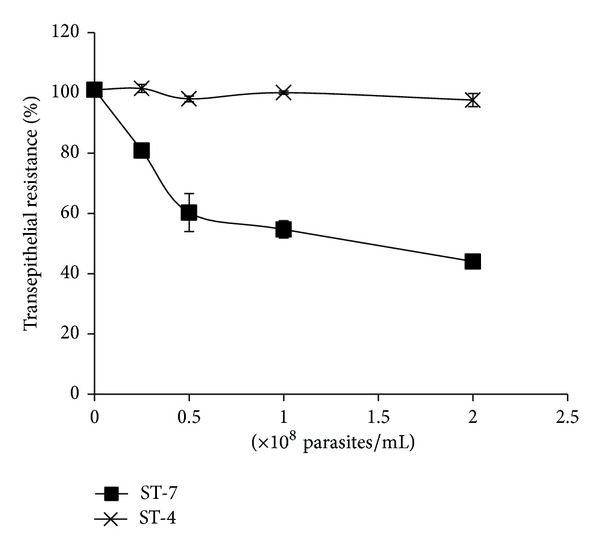
Dose-dependent effect of* Blastocystis* ST-4 and ST-7 on transepithelial resistant (TER) of Caco-2 cell monolayers. Confluent monolayers were coincubated with varying doses of ST-4 and ST-7 for 24 h. Compared to negative control,* Blastocystis *ST-7 induced a significant drop in Caco-2 TER at 0.25, 0.5, 1, and 2 × 10^8^ parasite/mL (*P* value < 0.01). Values are mean ± standard error (error bars) (*n* = 6).

**Figure 3:**
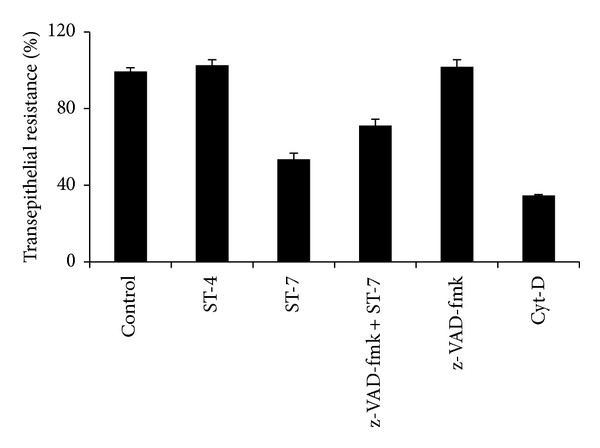
Effects of caspase inhibition on* Blastocystis *ST-7-induced decrease in transepithelial resistance (TER) of Caco-2 monolayers. Confluent monolayers of Caco-2 cells were coincubated for 24 h with ST-7 after pretreatment of cells with broad-spectrum caspase inhibitor z-VAD-fmk. Thereafter, TER was measured as described in Section 2. Pretreatment of Caco-2 cells with caspase inhibitor considerably rescued these cells from* Blastocystis *ST-7-induced effect (*P* value < 0.01). Cytochalasin D (Cyt-D) was used as a positive control in decreasing TER. Values are means ± standard error (error bars) (*n* = 6).

**Figure 4:**
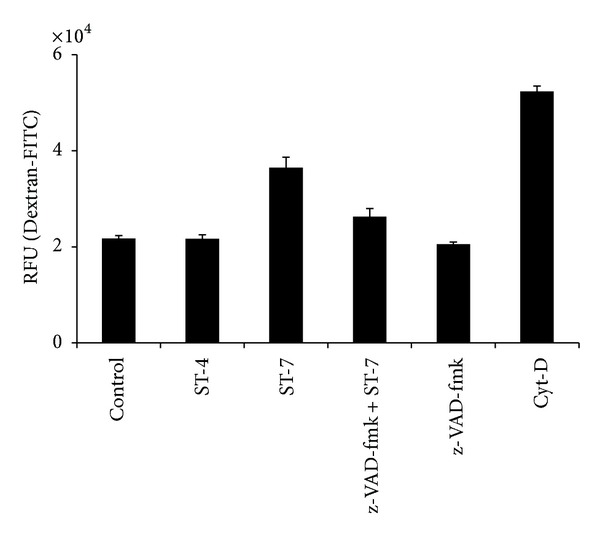
Flux measurement with FITC-conjugated Dextran. Confluent monolayers of Caco-2 cells were coincubated for 24 h with* Blastocystis *ST-4 or ST-7. Some monolayers were coincubated with ST-7 after pretreatment of cells with the broad-spectrum caspase inhibitor Z-VAD-fmk. Permeability was determined by measurement of Dextran-FITC fluxes across the monolayer as described in Section 2. A significant increase in the epithelial permeability can be noticed after incubation with ST-7, compared to negative control and ST-4 coincubation (*P* value < 0.01). Pretreatment of Caco-2 cells with caspase inhibitor significantly rescued these cells from* Blastocystis*-induced effect on permeability. Cytochalasin D (Cyt-D) was used as a positive control in inducing permeability increase. Values are means ± standard error (error bars) (*n* = 6).

*Blastocystis* ST-7, but not ST-4, induced a time-dependent drop in Caco-2 TER (Figure 1). A significant drop in Caco-2 TER was observed as early as after 3 h of coincubation (*P* value < 0.01). ST-7 also exhibited a dose-dependent effect on TER (Figure 2). The minimum dose of 0.25 × 10^8^/mL of ST-7 induced a significant drop in epithelial resistance (Figure 2). In order to confirm the parasite-mediated epithelial dysfunction suggested by drop in TER, we measured the flux of Dextran-FITC probe across Caco-2 monolayers (Figure 4). As expected, a significant increase in epithelial permeability was observed when 10^8^parasite/mL of ST-7 was coincubated with Caco-2 for 24 h (*P* value < 0.01) (Figure 4). On the other hand ST-4 did not cause a significant change in TER (Figures 1, 2, and 3) neither at the highest parasite dose (2 × 10^8^) (Figure 2) nor after the longest coincubation period (24 h) (Figure 1). ST-4 did not induce increase in epithelial permeability to FITC-conjugated Dextran either (Figure 4). This suggests a strain-dependent variation in parasite-mediated Caco-2 barrier disruption.

### 3.2. *Blastocystis *ST-7 Induces Early and Late Apoptotic Changes in Caco-2 Cells

**Figure 5:**
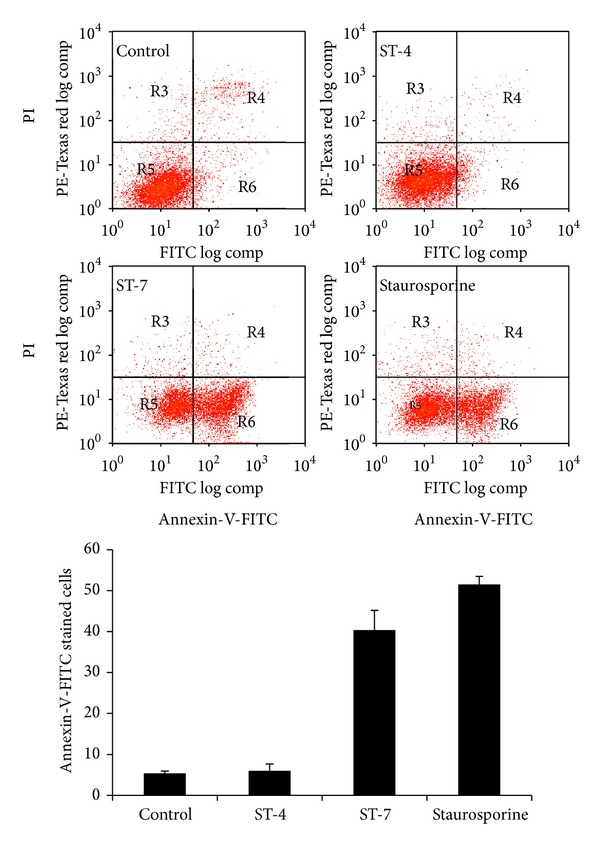
Flow cytometry analysis of annexin-V-FITC and propidium iodide staining. Representative dot plots of Caco-2 cells preincubated with culture media as negative control,* Blastocystis *ST-7, ST-4, and 0.5 *μ*M staurosporine as positive control. 2 × 10^4^ cells were analysed in each sample. Values represent mean ± standard error (error bars; *n* = 3). Caco-2 cells interacting with ST-7 exhibited significantly higher percentage of annexin-V^+^ and PI^−^ cells (lower right quadrant) compared to cells coincubated with ST-4 or culture media only (*P* value < 0.01). Values are means ± standard error (error bars) (*n* = 6).

*(i) PS-Flipping Indicated by Annexin-FITC Binding*. One of the early indicators of apoptosis is the flipping of phosphatidylserine (PS) molecules from the inner to outer leaflet of the plasma membrane. A 35- to 36-kDa molecule, annexin-V, binds to PS with high specificity in the presence of calcium. Viable, apoptotic, and necrotic cells can be distinguished when FITC conjugated-annexin-V is used in conjunction with PI. The lower right quadrants of the dot plots represent the apoptotic cell population, positive for annexin binding but PI negative (Figure 5). Upper quadrants represent necrotic cells due to permeability to PI (Figure 5). After interaction with* Blastocystis* ST-7, Caco-2 cells exhibited a significant rise in percentage of apoptotic cells compared to negative control and those interacting with ST-4 (*P* value < 0.01) (Figure 5). These findings are in agreement with a strain-dependent pathogenicity of the parasite. 

**Figure 6:**
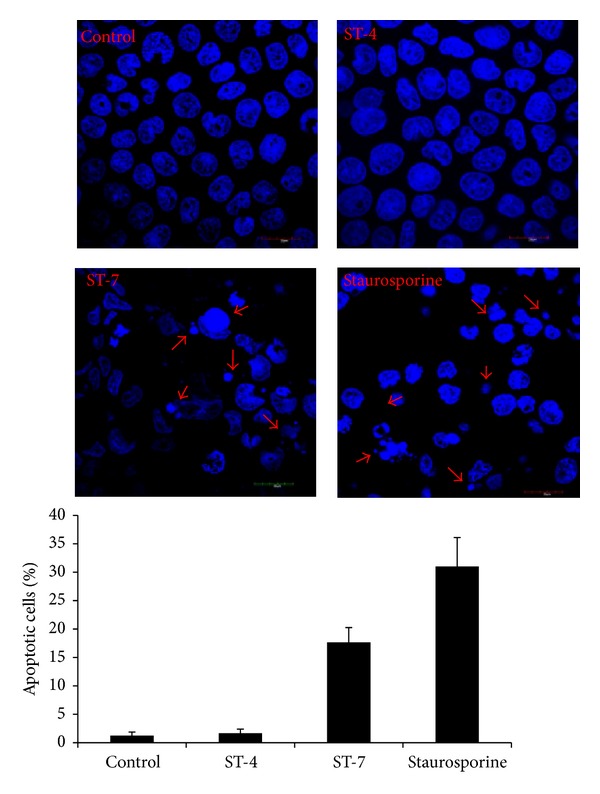
Representative fluorescence micrographs showing apoptosis of Caco-2 cells after DAPI staining. Cells were grown on glass coverslips and incubated for 24 h with culture media, ST-4, ST-7, or 0.5 *μ*M staurosporine as positive control. Cells coincubated with ST-7 and staurosporine exhibit nuclear fragmentation and condensation (arrow) typical of apoptotic cells. Histogram represents percentage of apoptotic cells after DAPI fluorescence assay. Caco-2 monolayers coincubated with ST-7 exhibited significantly higher percentage of apoptosis (*P* value < 0.05) compared to ST-4 and negative control. Values are means ± standard error (*n* = 6 per group). For each sample, ~100 cells were counted at 1000x magnification.

*(ii) Nuclear Fragmentation Indicated by Hoechst Staining [41].* One of the most distinctive features of apoptosis is morphological change in the nucleus, easily observed under florescence microscopy (Figure 6). After 24 h interaction with* Blastocystis *ST-7, nuclei of the Caco-2 cells exhibited nuclear condensation and fragmentation (Figure 6) typical of apoptotic cells. Significantly higher number of apoptotic cells was observed in membranes interacting with ST-7 compared to those with ST-4 (*P* value < 0.01) and negative control (*P* value < 0.01) (Figure 6).

### 3.3. *Blastocystis* ST-7 Induces Caspases 3 and 9 Activation in Caco-2 Cells

**Figure 7:**
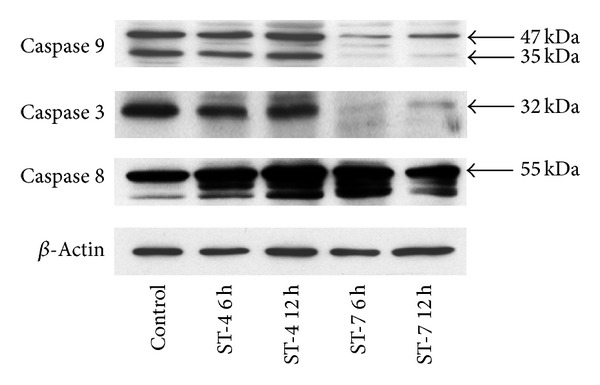
Western blot analysis of caspase activation (cleavage) in Caco-2 cells. Caco-2 cells were grown on cell-culture plates and harvested after coincubation with* Blastocystis* ST-4 and ST-7 for 6 and 12 h, as described in Section 2. Interaction with ST-7 resulted in loss of caspase 3 and caspase 9 bands suggesting activation, while caspase 8 remained unchanged. ST-4 did not cause any cleavage of caspases in Caco-2.

Caspases are proenzymes, which are activated by cleavage into active fragments in apoptotic cells. In this study, western blot analysis revealed that* Blastocystis* ST-7 coincubation with Caco-2, resulted in cleavage of Caco-2 caspases 3 and 9 (Figure 7). No cleavage of caspase 8 was observed even after 12 h interaction with ST-7 (Figure 7) suggesting that* Blastocystis* ST-7 induces Caco-2 programmed cell death by activation of the intrinsic apoptotic pathway. ST-4, as expected, did not activate any of the three caspases tested in this study (Figure 7).

### 3.4. *Blastocystis* ST-7 Induces Caspase-Dependent ZO-1 Rearrangement in Caco-2

**Figure 8:**
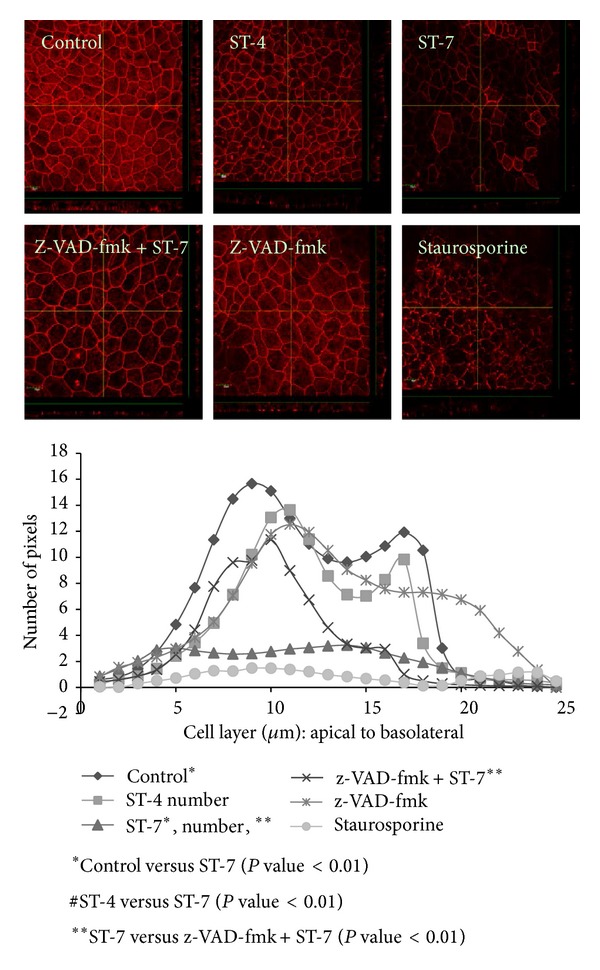
(a) Representative confocal micrographs illustrating ZO-1 distribution in Caco-2 monolayers. Monolayers were grown to confluence on poly-L-lysine treated coverslips. Caco-2 cells were then coincubated for 6 h with either* Blastocystis* ST-4 or ST-7. Some monolayers were treated with broad-spectrum caspase inhibitor z-VAD-fmk before coincubation with ST-7. Normal culture media and 0.5 *μ*M staurosporine were used as negative and positive controls, respectively. Compared to negative control, ST-7 treatment resulted in obvious reduction in ZO-1 apical localization in Caco-2 cell line. ST-4 did not alter ZO-1 integrity. Pretreatment with z-VAD-fmk rescued ST-7-induced ZO-1 changes in the epithelium. (b) Quantification of ZO-1 staining is shown as graphs. Each cell layer (1–25) corresponds to series of images from Z-stack sections taken at 1 *μ*m thickness through the cell monolayer shown in Figure 8. *X*-axis illustrates cell layers from apical to basolateral. *Y*-axis illustrates the number of pixels present over the entire area of image. Monolayers interacting with ST-7 and treated with staurosporine resulted in marked reduction in number of pixels in cell layers representing apical region, compared to ST-4 treated epithelium and normal control. Pretreatment of epithelium with broad-spectrum caspase inhibitor, z-VAD-fmk, resulted in inhibition of ST-7-induced ZO-1 changes in the monolayer. Results shown are mean of 4 separate Z-stacks for each treatment. (magnification: 600x).

**Figure 9:**
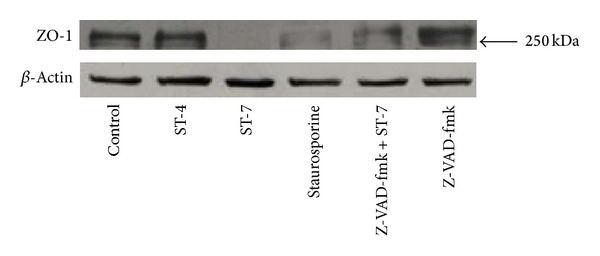
Western blot analysis of ZO-1 integrity in Caco-2 epithelium. Monolayers were grown on cell-culture plates and harvested after coincubation with* Blastocystis* ST-4, ST-7, normal growth media, and 0.5 *μ*M staurosporine. Monolayers were also treated with broad-spectrum caspase inhibitor z-VAD-fmk before incubation with ST-7. After coincubation with ST-7 and staurosporine for 6 h, loss of ZO-1 band was observed. The ZO-1 band remained unchanged in untreated Caco-2 cells and those interacting with ST-4. Caspase inhibition resulted in rescue of ST-7-induced loss of ZO-1.

Compromise of epithelial barrier function, as observed in this study, is often associated with alterations of tight junction proteins. In this study, we observed that* Blastocystis* ST-7 induced an alteration in tight junction protein complex in conjunction with epithelial barrier dysfunction (Figures 8 and 9). Confocal micrographs suggest that exposure of Caco-2 monolayer to ST-7 induced a significant drop in anti-ZO-1 antibody binding to the apical junctional ring of epithelium (*P* value < 0.01) (Figure 8). ST-4 and negative control, on the other hand, had no effect on epithelial ZO-1 (Figure 8). Furthermore, western blot analysis showed that interaction with ST-7 resulted in a loss of Caco-2 ZO-1 band at 250 kDA (Figure 9). Interaction with ST-4 and negative control had no effect on ZO-1 distribution in Caco-2 (Figures 8 and 9). Interestingly, inhibition of host caspases by z-VAD-fmk prevented ST-7-induced ZO-1 changes (Figures 8 and 9).

### 3.5. Inhibition of Caco-2 Caspases Prevented* Blastocystis* ST-7-Induced Epithelial Barrier Dysfunction

It has been reported previously that host epithelial dysfunction induced by luminal parasites is caused by increased apoptosis in enterocytes. In this study, we observed that inhibition of host caspases by broad-spectrum caspase inhibitor, z-VAD-fmk, significantly prevented ST-7-induced TER drop (*P* value < 0.01) (Figure 3) and inhibited parasite-induced increase in permeability (*P* value < 0.01) (Figure 4) of Caco-2 monolayers. These findings (Figures 3 and 4), in conjunction with inhibition of ST-7-induced ZO-1 alteration (Figures 8 and 9) by z-VAD-fmk, suggested a role of parasite-induced apoptosis in epithelial barrier dysfunction.

## 4. Discussion

In this study, using two clinically relevant* Blastocystis *isolates, we reported that a* Blastocystis *ST-7 isolate recovered from a human patient-induced epithelial barrier dysfunction in Caco-2 human epithelial cell-line, while a ST-4 isolate recovered from rat host did not induce enterocyte pathology. The intestinal epithelium serves as the body's first line of defense against luminal contents comprising of a diverse flora including pathogenic and nonpathogenic prokaryotes and eukaryotes [33]. Intestinal epithelial cells or enterocytes regulate the back and forth flow of contents between the gut lumen and subepithelial tissue [33]. Dysfunction of this barrier is the cause of a wide range of human diseases [28].* Blastocystis*-induced compromise of human epithelial barrier, observed in our study, reinforces its status as a human pathogen.

We are also reporting for the first time that* Blastocystis* causes apoptosis in human epithelial cells. Apoptosis is a preprogrammed mechanism of clearing up unwanted cells by the body [42] without overtly stimulating a host immune response [36]. Cells undergo apoptosis even under physiological conditions, but several pathogens including parasites have evolved mechanisms to disrupt this machinery, by either upregulating [38, 43] or downregulating [44] it for their survival in the host.* Blastocystis *ST-7 induced both early and late hallmarks of apoptosis, that is, PS-externalization and nuclear fragmentation, respectively, in human enterocytes. Increased enterocyte apoptosis in some cases is also proposed to be a host response to infection by increasing the cell turnover in order to rid the body of the infected cells [45]. Mucosal sloughing reported during* Blastocystis* infections [46] might be a result of this increased turnover. Anti-inflammatory effects of apoptosis are also well recognized [47]. The relatively moderate level of apoptosis induced by* Blastocystis *in intestinal epithelium is similar to the programmed cell death of enterocytes when exposed to* Cryptosporidium *[48] and* Giardia *[38]. It is suggested that upregulation of host cell apoptosis might be a reason for lack of overt host inflammatory response during parasitic infections [38, 48] and might assist them in colonizing the hostile host environment. Although further investigation is needed in this area, the absence of obvious gut inflammatory changes in* Blastocystis*-infected hosts might be due to the ability of the parasite to downregulate host inflammatory response by induction of enterocyte apoptosis.

This is also the first study reporting that* Blastocystis *induces epithelial apoptosis by activation of the intrinsic pathway. Caspase 3 activation lies at the center of the caspase-mediated apoptosis [42]. It is preceded by activation of either intrinsic pathway, involving mitochondrial injury and caspase 9 activation, or extrinsic pathway due to Fas/FasL receptor-mediated caspase 8 activation. Parasites have evolved complex mechanisms to activate epithelial caspase 3.* Giardia* activates extrinsic as well as intrinsic apoptotic pathways [49].* Entamoeba* on the other hand does not require either caspase 8 or caspase 9 for enterocyte caspase 3 activation [50]. In an earlier study, we reported that* Blastocystis* ST-4 induced rat epithelial apoptosis by caspase 3 activation [39]. There is no data available suggesting a similar outcome in human epithelium. Upstream pathways involved in caspase 3 activation by the parasite are not known either. In this study, the rodent strain had no effect on Caco-2 cell line, but ST-7, isolated from human host, induced caspase 3 activation.* Blastocystis *unlike* Giardia *or* Entamoeba *only activated caspase 9. A recent study suggested the involvement of Rho kinases in selective activation of caspase 9, leading to apoptosis [51]. A role of Rho kinase has been suggested in* Blastocystis*-induced breakdown of host epithelial barrier function and cytoskeletal rearrangement [22]. Although more data is required, selective activation of caspase 9 by* Blastocystis *in this study might be a result of epithelial Rho kinase modulation by the parasite. Further understanding of the unique cellular mechanisms employed by* Blastocystis* to induce host cell apoptosis might help us to develop targeted therapeutics to prevent parasite-induced host pathology.

Tight junctions are key regulators of epithelial barrier function [33, 52]. ZO-1 is an important component of apical junctional complex, anchoring tight junctions to actin cytoskeleton [33]. Our data shows that* Blastocystis* ST-7 induces rearrangement of ZO-1 in intestinal epithelium. Several parasites are known to cause ZO-1 alterations by a wide array of mechanisms [33, 53, 54].* Acanthamoeba* activates Rho/Rho-kinase pathway [53], whereas* Giardia* utilizes myosin light chain kinase [54] and caspases [38] to induce changes in ZO-1 organization. Apoptosis also plays a diverse role in the modulation of epithelial barrier function and tight junction reorganization. On one hand, enterocyte apoptosis ensures that epithelial barrier remains sealed [52], while in other cases induction of apoptosis is employed by pathogens to increase epithelial permeability [38] and cause host pathology [37]. Inhibition of host caspases resulted in prevention of* Giardia-*induced modulation of epithelial permeability and ZO-1 organization [38] while, inhibition of caspase-mediated apoptosis in enteropathogenic* E. coli* (EPEC) infections did not prevent the epithelial ZO-1 alterations [55]. In a recent study with rodent epithelium, pretreatment of host epithelium with pan-caspase inhibitor z-VAD-fmk did not rescue* Blastocystis *ST-4-induced epithelial barrier dysfunction [39]. In the current study, z-VAD-fmk treatment of epithelium significantly inhibited* Blastocystis* ST-7-induced epithelial barrier compromise. Parasite-induced ZO-1 alteration was also prevented significantly by host caspase inhibition, reiterating that role of enterocyte apoptosis in parasite- induced epithelial barrier dysfunction. Interestingly, changes in ZO-1 were also shown to be prevented by Rho kinase [22], again raising the question of the role of Rho kinase in caspase-mediated apoptosis.

We also observed for the first time a strain-dependent variation in* Blastocystis-*induced epithelial barrier compromise.* Blastocystis* ST-4 did not induce an increase in epithelial permeability, enterocyte apoptosis, or ZO-1 rearrangement in Caco-2 cells. We have also similarly observed that ST-7 and not ST-4 induced apoptosis in HT-29 cells, another transformed human intestinal epithelial cell line (results not shown). Several studies have suggested strain-dependent differences in* Blastocystis* virulence [8, 25]. Strain-to-strain variation in virulence is not unique to* Blastocystis* and it has been observed in intestinal parasites such as* Giardia*,* Cryptosporidium, *and* Entamoeba *[38, 56, 57], providing a plausible explanation for the large number of asymptomatic carriers of these pathogens [58, 59]. This might also explain frequent reports of asymptomatic* Blastocystis *carriers. In a recent study [26], we reported that* Blastocystis *exhibits a strain-dependent variation in the activity of cysteine proteases, potential parasite virulence factors [22, 27]. ST-7 of the parasite, reported to have higher cysteine protease activity [26], caused epithelial barrier dysfunction in the current study, while ST-4 with comparatively lower cysteine protease activity [26] did not. Although more data is needed, these findings suggest a possible association between parasite cysteine protease activity and its ability to induce epithelial barrier dysfunction. Interestingly, ST-4 caused epithelial barrier dysfunction in rat epithelium [39], suggesting that this strain exhibits host specificity in its ability to induce epithelial barrier compromise, as observed in* Cryptosporidium* infections [56]. Although human ST-4 infections are commonly associated with intestinal symptoms, its inability to induce epithelial barrier compromise in this study suggests that ST-4 might induce human pathology by some other mechanisms [60, 61].

To conclude, this is the first study to show that* Blastocystis *ST-7, a parasite subtype recovered from a human patient, induced enterocyte-apoptosis by activating caspases 3 and 9, suggesting the involvement of the intrinsic apoptotic pathway in pathogenesis. We also showed that this cytopathic human isolate of* Blastocystis* (ST-7) caused rearrangement of ZO-1 protein. Inhibition of host caspases prevented parasite-induced epithelial barrier dysfunction as well as ZO-1 rearrangement, suggesting the role of caspase-dependent enterocyte apoptosis in host epithelial barrier dysfunction induced by* Blastocystis*. Furthermore, the inability of rodent subtype ST-4 to induce any changes in Caco-2 provides evidence of host specificity and strain dependency in* Blastocystis*-induced human epithelial pathology. The strain-to-strain variation in parasite virulence is a plausible explanation for the large number asymptomatic human carriers of* Blastocystis*.
